# Dominion of Canada

**Published:** 1896-09

**Authors:** 


*Dominion of Canada.* The Dominion of Canada is now working under a Parliamentary act which is almost prohibitive in its requirements regulating the importation of horses. The certificates of no less than three veterinarians are required; one certifying to the health of the horse, another the public health of the district from which the animal was shipped, and the third the public health of the port of embarkation. The second certificate would be difficult to give, and might be of little value when given, for we have no sanitary police laws which keep the profession posted as to the existence of contagious or infectious disease among our live-stock.

---

A match race has been made between W. W. Hamilton, the bicyclist, and the horse "Joe Patchen," best two in three.

Veterinarian G. Howard Davis, of Millbrook, N. Y., has returned from England, and, in addition to some choice sheep, he has brought home fourteen goats, the milk of which he proposes to introduce into New York. The doctor believes that the freedom of these animals from tuberculosis will develop a good market for their milk among infants and invalids. Goats' milk is very rich and nutritious. Dr. Davis was a large exhibitor of Shropshire sheep at the New York State Fair.

